# Nutrient Composition Promotes Switching between Pellicle and Bottom Biofilm in *Salmonella*

**DOI:** 10.3389/fmicb.2017.02160

**Published:** 2017-11-07

**Authors:** Sonia Paytubi, Cintia Cansado, Cristina Madrid, Carlos Balsalobre

**Affiliations:** Section of Microbiology, Virology and Biotechnology, Department of Genetics, Microbiology and Statistics, University of Barcelona, Barcelona, Spain

**Keywords:** biofilm, *Salmonella*, adenylate cyclase, amino acids, nutrient composition studies, curli, cellulose

## Abstract

*Salmonella* is one of the most frequently reported causes of foodborne illness worldwide. Non-typhoidal serovars cause gastroenteritis in humans. *Salmonella* can grow on surfaces forming biofilms, contributing to its persistence since biofilms are difficult to eradicate due to the high resistance to antimicrobials and disinfectants. It has been described that there are two crucial biofilm promoting factors in *Salmonella*: curli and cellulose. The expression of both factors is coordinately regulated by the transcriptional regulator CsgD. Most biofilm studies of *Salmonella* have been performed by growing bacteria in low osmolarity rich medium and low temperature (25°C). In such conditions, the biofilm is formed at the air–liquid interface (pellicle biofilm). Remarkably, when *Salmonella* grow in minimal medium, biofilm formation switches from the air–liquid interface to the solid–liquid interface (bottom biofilm). In this report, the switching between pellicle and bottom biofilm has been characterized. Our data indicate that curli, but not cellulose, is crucial for the formation of both kinds of biofilms. In minimal medium, conditions promoting formation of bottom biofilm, a high transcriptional expression of *csgD* and consequently of the genes involved in the synthesis of curli and cellulose was detected. The nutritional status of the cells seems to be pivotal for the spatial distribution of the biofilms formed. When bacteria is growing in minimal medium the addition of amino acids downregulates the expression of *csgB* and causes the switch between bottom and pellicle biofilm. The crosstalk between general metabolism and biofilm formation is also highlighted by the fact that the metabolic sensor cAMP modulates the type of biofilm generated by *Salmonella*. Moreover, cAMP regulates transcriptional expression of *csgD* and stimulates pellicle biofilm formation, suggesting that the physiological conditions define the type of biofilm formed by *Salmonella*. The consequences of the switching between pellicle and bottom biofilm during either infection or survival in natural environments remain undercover.

## Introduction

Microbial communities attached to surfaces and embedded in a self-produced extracellular polymeric matrix are defined as biofilms. Those complex structures are the principal mode of microbial growth in nature (Steenackers et al., 2012). Development of a biofilm starts when a motile bacterial cell approaches and adheres reversibly to a surface. The biofilm formation process continues when the attached cells form a microcolony. The subsequent synthesis of an extracellular matrix will support the formation of a mature three-dimensional biofilm. Cells within the biofilm can undergo controlled lysis and escape from the microbial community promoting biofilm dispersal (O’Toole et al., 2000). As bacterial biofilms may provide a reservoir of pathogenic bacteria, they represent a threatening concern by increasing the risk of microbial contamination. Bacterial biofilms cause critical problems in terms of public health and economical lost (Shi and Zhu, 2009). Bacteria within biofilms become recalcitrant and highly resistant to antimicrobials and disinfectants. Consequently, when bacteria are part of a biofilm are extremely difficult to eradicate (O’Toole et al., 2000; Bridier et al., 2011).

*Salmonella* biofilms can be found attached to both biotic and abiotic surfaces. Well documented biotic surfaces are gallstones and animal epithelial cells (reviewed in Steenackers et al., 2012). Moreover, it has been described that *Salmonella* can form biofilm on plant surfaces, being the consumption of contaminated vegetables the cause of numerous recent outbreaks (Heaton and Jones, 2008). *Salmonella* can also form biofilm on a broad range of abiotic surfaces such as glass, plastic, rubber, cement, and stainless steel, which are materials of common use in food processing industries. It has been estimated that biofilm contaminated surfaces are a relevant mode of transmission of pathogens and spoiling bacteria during food processing (Frank and Chmielewski, 2001). The first report on foodborne bacterial biofilm describe the ability of *Salmonella* to adhere to food surfaces (Duguid et al., 1966). *Salmonella* spp. non-typhoidal is the causal agent of salmonellosis, a foodborne disease with high incidence worldwide. In United States, *Salmonella* causes up to 1,200,000 cases of gastroenteritis, with 23,000 hospitalizations and 450 deaths every year (Scallan et al., 2011). The high persistence of non-typhoidal *Salmonella* relies on its ability to form biofilms (Steenackers et al., 2012).

The widely used laboratory set-up to study *Salmonella* biofilms consists in growing bacteria in rich medium with low osmolarity, at low temperature (25–28°C) and static incubation. Under those conditions *Salmonella* forms a biofilm at the air–liquid interface, known as pellicle. Earlier observations from us and other authors showed that the type of biofilm formed by *Salmonella* varies depending on the media composition, since in minimal media a solid–liquid interface biofilm (bottom) is observed (Römling and Rohde, 1999; Paytubi et al., 2017). In this report, this phenomenon was further studied. Our data indicate that in minimal medium, conditions that promote bottom biofilm, there is higher expression than in rich medium of the biofilm regulator *csgD* and subsequently of curli and cellulose. The influence of nutrient composition in the spatial distribution of the biofilm generated by *Salmonella* has been shown since the presence of amino acids promotes the formation of pellicle biofilm. Moreover, the metabolic sensor cAMP modulates biofilm location by stimulating pellicle biofilm formation. Expression studies indicate that cAMP causes a drop in the expression of the biofilm promoting factors by modulating the levels of CsgD.

## Materials and Methods

### Bacterial Strains and Culture Media

The *Salmonella enterica* serovar Typhimurium (*S.* Typhimurium) wild type (wt) strain SV5015 is a His^+^ derivatives of the mouse virulent strain SL1344 (*hisG64*, *rpsL*) (Hoiseth and Stocker, 1981; Vivero et al., 2008). All strains and plasmids used in this work are described in Supplementary Table S1.

Cultures were routinely grown in LB-agar plates (10 g/l NaCl, 10 g/l tryptone, 5 g/l yeast extract, and 15 g/l agar). For biofilm, β-galactosidase assays, and calcofluor determinations, cultures were grown in colonization factor antigen (CFA) medium (10 g/l casamino acids, 1.5 g/l yeast extract, 0.4 mM MgSO_4_, and 0.4 mM MnCl_2_) (Suzuki et al., 2002) or E minimal medium (MM). MM was prepared by diluting a stock of Vogel-Bonner salts 50× to a final concentration of 1× (0.8 mM MgSO_4_, 9.5 mM citrate, 57 mM K_2_HPO_4_, and 17 mM NaNH_4_HPO_4_) (Vogel and Bonner, 1956). When indicated, media was supplemented with NaCl to reach a final concentration of 10 g/l.

Kanamycin (Km, 50 μg/ml), chloramphenicol (Cm; 25 μg/ml), and X-Gal (40 μg/ml) were added when required.

For complementation assays, 5 mM cAMP was prepared in CFA and sterile filtered.

To test the effect of different carbon sources on minimal medium, M9 medium (Sambrook and Russell, 2001) was used and supplemented with the indicated sterile-filtered carbon source to reach a final concentration of 0.2% (w/v).

Twenty amino acids mixture was prepared at the following concentrations (Neidhardt et al., 1977): 0.1 mM cysteine and tryptophan; 0.2 mM methionine, histidine, and tyrosine; 0.4 mM isoleucine, asparagine, phenylalanine, aspartate, arginine, lysine, threonine, and proline; 0.6 mM glutamine, valine, and glutamate; 0.8 mM alanine, leucine, and glycine; 10.0 mM serine.

### Construction of Mutations and *lacZ* Gene Fusions

Chromosomal mutants were generated by one-step gene replacement by homologous recombination (Datsenko and Wanner, 2000). Open reading frames were replaced by either a Km or Cm resistance marker. Briefly, primers containing homology extensions to 5′ and 3′ of the gene to be replaced and sequences flanking the antibiotic marker encoded in either plasmid pKD4 (Km) or pKD3 (Cm) were used to amplify the antibiotic resistance cassette. Primers used in this work are listed in Supplementary Table S2. The purified PCR product was *Dpn*I digested and electroporated into *S.* Typhimurium SV5015 harboring plasmid pKD46. Recombinant clones were selected in LB-agar plates containing either Km or Cm. For transcriptional studies, *lacZ* fusions were integrated into the gene of interest as follows (Ellermeier et al., 2002). First the antibiotic marker was removed expressing the FLP protein encoded in plasmid pCP20 that promotes site-specific recombination between the FRT sites contained in the antibiotic resistance cassette. Next, plasmid pKG136 was electroporated into the resulting strains and again FLP-mediated recombination was achieved by electroporating plasmid pCP20. Recombinant clones were selected in LB-agar plates containing Km and X-Gal.

Mutant alleles were transducted into a novel strain background by phage P22 HT/int4 (Sternberg and Maurer, 1991). Transductants were streaked twice on EBU LB-agar plates [0.25% (w/v) glucose, 0.25% (w/v) K_2_HPO_4_, 0.0125 g/l Evans blue, and 0.0250 g/l fluorescein] (Maloy and Stewart, 1996) supplemented with the corresponding antibiotics. All constructed mutants were PCR-verified with control primers located in the genes flanking the deleted open reading frame (Supplementary Table S2).

### Biofilm Formation, Detection, and Quantification

Liquid cultures were grown using sterile, flat-bottomed, 24-well polystyrene plates (Nunc). For biofilm formation assays each well plate was filled with 1 ml of culture media containing the *Salmonella* inoculum. The optimum inoculum per well was achieved by diluting a *Salmonella* suspension to an OD_600__nm_ of 0.02. Plates were incubated for 72 h at 25°C in static conditions inside a plastic bag with wet cellulose paper to maintain the correct humidity levels. For visual detection of biomass attached to the plastic surface and for quantitative measurement of the biofilm biomass, crystal violet (CV) staining was performed. CV binds to negatively charged molecules and is frequently used to stain and quantify total biofilm biomass containing bacteria and extracellular polymeric substances (Stepanovic et al., 2000). CV staining protocol was performed as described by Paytubi et al. (2017) with minor modifications. Briefly, plates were rinsed twice with distilled water and biofilms were fixed by heating at 80°C for 30 min. Then, 2 ml of a 1% (w/v) CV solution, was added to the wells and incubated for 15 min, rinsed with water and air-dried. One milliliter of acetic acid 30% (v/v) was added to the wells and the plate was shaken gently to make sure the CV was totally solubilized. The OD_570__nm_ of the resulting solution was determined. As a negative control, 1 ml of the corresponding media without inoculum was used. The signal detected in the negative control was subtracted from the values obtained for all the samples.

### β-Galactosidase Assay

β-Galactosidase assays were performed as described by Miller (1992). Briefly, cultures were routinely grown as described for biofilm formation (72 h at 25°C on a 24-well polystyrene plate). After 72 h, the OD_600__nm_ of the homogenized cultures was determined and 0.1 ml was added to 0.9 ml of Z buffer (60 mM Na_2_HPO_4_, 40 mM NaH_2_PO_4_, 10 mM KCl, 1 mM MgSO_4_, and 50 mM 2-mercaptoethanol, pH 7) in borosilicate glass tubes. After that, 50 μl of chloroform and 25 μl of SDS 0.1% (w/v) were added and the mixture was intensively vortexed for 15 s. Samples were incubated at 28°C during the entire reaction assay that was initiated upon addition of 0.2 ml of ONPG (4 mg/ml) in Z buffer and terminated by the addition of 0.5 ml of 1 M Na_2_CO_3_. After 5 min, OD_420__nm_ and OD_550__nm_ of the reaction mixtures was determined and β-galactosidase activity (Miller Units) was calculated using the following formula.

Miller Units = 1000 × (OD_420_ - 1.75 × OD_550_)/[volume (ml) × time (min) × OD_600_].

Enzymatic determinations were performed in duplicates of at least three independent experiments and the mean values are plotted with standard deviations.

### Cellulose Quantification

Qualitative assessment of bacterial cellulose production was determined on calcofluor (CF) agar plates. Five microliters of a bacterial suspension from fresh LB-agar plates in PBS (OD_600__nm_ of 2.0) were spotted on CFA-agar plates supplemented with 0.2 mM calcofluor white (fluorescent brightener 28, Sigma). Plates were incubated at 25°C for 72 h, and fluorescence was observed under a UV light source.

Cellulose production was quantified by measuring the calcofluor bound to cells (Pérez-Mendoza et al., 2014). Bacterial suspensions obtained as above were diluted up to an initial OD_600__nm_ of 0.005 into 50 ml flasks containing 10 ml of CFA or MM, supplemented with CF (0.1 mM), and incubated at 25°C under shaking conditions (100 rpm) for 72 h. Cultures were centrifuged (10 min at 3,000 × *g*) to eliminate broth and unbound CF and cells were resuspended in 2 ml of water and disposed in 96-well plates. Fluorescence measurements were performed in a Cary Eclipse Fluorescence Spectrophotometer (excitation 360 nm, emission 460 nm). The results were normalized according the number of cells and expressed as relative arbitrary fluorescence units. Data are mean values of quadrupled determinations plotted with standard deviations. Bacterial suspensions were also observed under UV light.

### Rdar Morphotype

The rdar morphotype was judged visually on Congo red agar plates. Five microliters of a bacterial suspension in water (OD_600__nm_ of 5.0) from a fresh LB-agar plate were spotted onto LB agar plates without NaCl and complemented with Congo red (40 mg/l) and Coomassie brilliant blue G-250 (20 mg/l). Plates were incubated at 25°C for 96 h and development of the colony morphology and color was analyzed.

### Statistical Analysis

Differences between average values were tested for significance by performing an unpaired two-sided Student’s *t*-test. The levels of significance of the resulting *p*-values are reported by the following symbols: ^∗^*p* < 0.05, ^∗∗^*p* < 0.005, and ns, non-significant.

## Results And Discussion

### *Salmonella* Biofilm Spatial Distribution Varies in Different Culture Media

**FIGURE 1:**
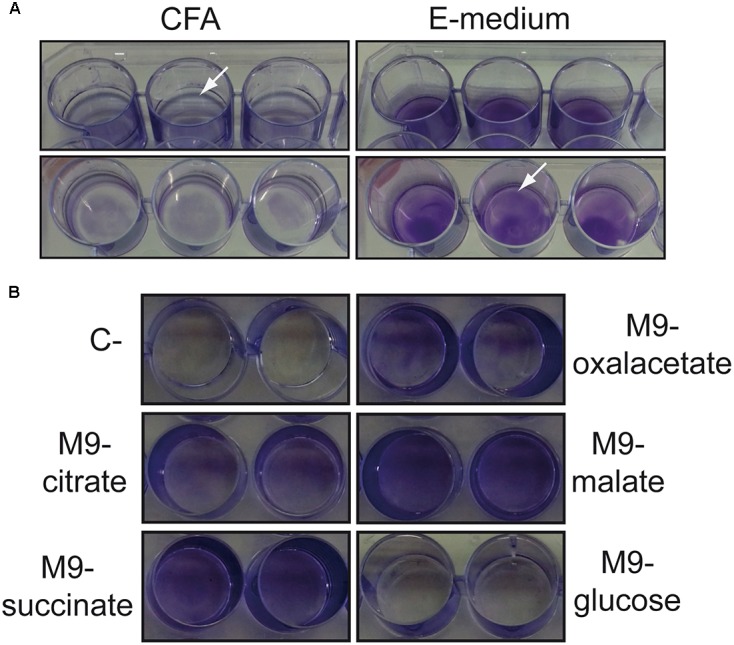
Types of biofilms formed by *S.* Typhimurium SV5015 grown in different culture media. **(A)** Biofilm formed when grown in CFA (pellicle) or E medium (bottom) on a 24-well polystyrene plate. **(B)** Biofilm formed when grown in M9 with the indicated carbon sources on a 24-well polystyrene plate. Biofilms attached to the plastic surface were stained with CV after 72 h incubation at 25°C. The arrows show the satined biofilms.

*Salmonella* biofilm studies have been routinely performed by growing bacteria in low osmolarity rich medium at low temperature (25°C) with static incubation. Under such conditions, *Salmonella* forms a biofilm at the air–liquid interface (pellicle biofilm). Recently, we described that *S. enterica* serovar Enteritidis strain 3934 forms a pellicle biofilm when grown in CFA (rich medium) but forms a solid–liquid interface biofilm (bottom biofilm) when MM (E minimal medium) is used. This phenomenon was also observed in other strains of *Salmonella* from serovars Typhimurium and Enteritidis (Paytubi et al., 2017). To characterize the mechanisms involved in the differential spatial distribution of the biofilm attending to the medium composition, *S.* Typhimurium strain SV5015, that can be easily genetically manipulated, was used. The ability to form biofilm on a polystyrene surface after growing for 72 h at 25°C in either CFA or MM was monitored by CV staining and visual inspection (**Figure 1A**). In CFA, SV5015 forms a pellicle biofilm which is detected as a blue ring on the solid surface exposed to the air–liquid interface. Contrarily, in MM, no pellicle biofilm was observed. Instead, a clear bottom biofilm was detected along the plastic surface in contact with the culture medium.

The MM used in those experiments, E medium, contains citrate as a carbon source. To clarify whether the differential biofilm distribution observed is specifically due to the presence of citrate, biofilm formed by SV5015 in minimal medium (M9) containing different carbon sources was monitored (**Figure 1B**). The bottom biofilm was detected in the presence of citrate, succinate, oxaloacetate, and malate as carbon sources. However, in the presence of glucose no biofilm was observed. The presence of glucose has been reported to inhibit biofilm formation in *Salmonella*, presumably by repressing a component required in the early stages of bacterial adhesion (White et al., 2010).

**FIGURE 2:**
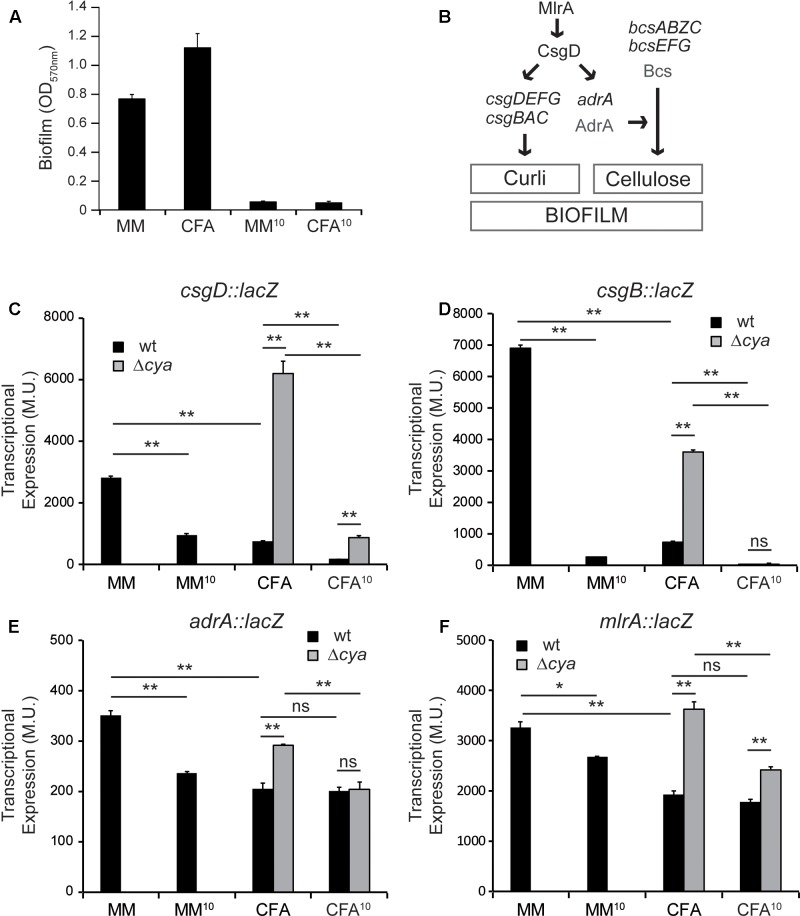
Differential expression of biofilm promoting factors. **(A)** Effect of minimal media (MM) and rich media (CFA) with or without salt on biofilm formation. **(B)** Simplified model of the regulatory network regulating curli and cellulose in *S. enterica*. **(C–F)** Effect of different media and *cya* mutation on the expression of biofilm effectors and regulators *csgD*, *csgB*, *adrA*, and *mlrA*, in cultures grown in the indicated media for 72 h at 25°C on a 24-well polystyrene plate. ^∗^*p* < 0.005, ^∗∗^*p* < 0.005, ns: non-significant.

Total biofilm biomass of cultures grown in the two assessed culture media (MM and CFA) was monitored by quantification of the CV retained in the biofilm attached to the plastic surface (**Figure 2A**). Since it is known that increasing osmolarity by the addition of NaCl causes a concomitant drop in the ability of *Salmonella* to form biofilm (Römling et al., 1998, 2000), negative controls for biofilm formation were performed using cultures grown in both MM and CFA supplemented with 10 g/l of NaCl (MM^10^ and CFA^10^, respectively). Despite of producing different type of biofilm in MM (bottom) and CFA (pellicle), both types of biofilms were efficiently detected by CV quantification. As expected, a negligible amount of biomass was detected in cultures grown in high osmolarity media.

### Transcriptional Expression of Genes Involved in Curli and Cellulose Synthesis Are Upregulated in Minimal Medium

Although there are many different components in the extracellular matrix present in *Salmonella* biofilms, two appendages—the amyloid fiber curli and the glycan polymer cellulose—are considered crucial promoting factors for *Salmonella* biofilm development (Gerstel and Römling, 2001). As summarized in **Figure 2B**, both curli subunits and its direct regulatory factor, CsgD, are encoded in two divergently transcribed operons *csgBAC* and *csgDEFG*. Additionally, the synthesis of cellulose requires the expression of two divergent operons *bcsABZC* and *bcsEFG*. The expression of the *bcs* genes is constitutive. However, cellulose production occurs when the diguanylate cyclase AdrA, that synthesizes the c-di-GMP which in turn activates the cellulose synthase, is expressed (Zogaj et al., 2001). Expression of the two biofilm-promoting factors is co-regulated, since the response regulator CsgD stimulates expression of both *adrA* and the genes coding for the structural curli subunits, *csgBAC*. Moreover, transcriptional expression of *csgD*, which is tightly regulated in response to different environmental stimuli, is activated by the regulator MlrA (Brown et al., 2001).

The transcriptional expression of the main components of the gene cascade that control biofilm formation in *Salmonella*—*csgD*, *csgB*, *adrA*, and *mlrA*—was studied using chromosomal *lacZ* transcriptional fusions. High osmolarity cultures were used as control of non-permissive conditions for biofilm formation (as shown in **Figure 2A**). The expression of *csgD*, the major regulator that coordinates expression of biofilm promoting factors, is severely affected by the composition of the culture media (**Figure 2C**, black bars). *csgD* transcriptional expression is up to 3.7-fold higher in MM than in CFA and, as expected, it drops considerably in both MM^10^ and CFA^10^ (Vidal et al., 1998; Prigent-Combaret et al., 2001; Brombacher et al., 2006). Accordingly, *csgB* follows the same expression pattern (**Figure 2D**, black bars). Transcription of *csgB* is also drastically affected by the composition of the culture media, being up to 9.4-fold higher in MM than in CFA, suggesting that in MM there is more production of curli than in CFA. The expression profile of *adrA*, the diguanylate cyclase that stimulates cellulose production, is slightly different to that described for *csgB* and *csgD* (**Figure 2E**, black bars). In MM there is also higher expression of *adrA* than in CFA suggesting that in MM cellulose production is stimulated. Remarkably, in rich medium (CFA), the *adrA* expression levels are very similar to those detected in non-permissive conditions for biofilm formation (high osmolarity) suggesting that *adrA* expression and cellulose production are very low in rich medium. Different CsgD binding patterns for *csgB* and *adrA* promoters have been described, thus suggesting the existence of different transcriptional activation mechanisms by CsgD for both genes (Zakikhany et al., 2010; Steenackers et al., 2012).

It has been described that *csgD* expression is stimulated by MlrA (Brown et al., 2001). The transcriptional expression of *mlrA* was monitored in the different culture media and an expression profile similar to the described for *adrA* was observed when comparing MM and CFA (**Figure 2F**, black bars). To determine whether the described *csgD* regulation was mediated by MlrA, experiments using an *mlrA* mutant were performed (Supplementary Figure S1). The expression level of *csgD* is lower in the *mlrA* mutant strain as compared to wild type (wt) (compare **Figure 2C** and Supplementary Figure S1), indicating that MlrA is required for full *csgD* expression. Furthermore, the *csgD* expression pattern in MM and CFA in the *mlrA* mutant is similar to that described in wt, indicating that MlrA is not required for the regulation of *csgD* by the composition of the culture media.

From those results it can be concluded that growth conditions that promote bottom biofilm formation correlate with high expression of *csgD* and, consequently, increased expression of *csgB* and *adrA*. On the contrary, low curli and cellulose production is expected when growing under conditions promoting pellicle formation attending to the low expression levels of *csgB* and *adrA* detected as compared with non-permissive conditions for biofilm formation (high osmolarity).

Previous findings suggest that biofilm formation by *Salmonella* is promoted in environments with low nutrient concentration and *csgD* expression is induced during nutrient starvation (Gerstel and Römling, 2001; Stepanovic et al., 2004). Our report indicates that nutrient composition, regardless of a possible effect in the total biofilm biomass, has a relevant effect to define the spatial location of the biofilm generated.

### Curli But Not Cellulose Is Essential for the Development of Both Types of Biofilm

**FIGURE 3:**
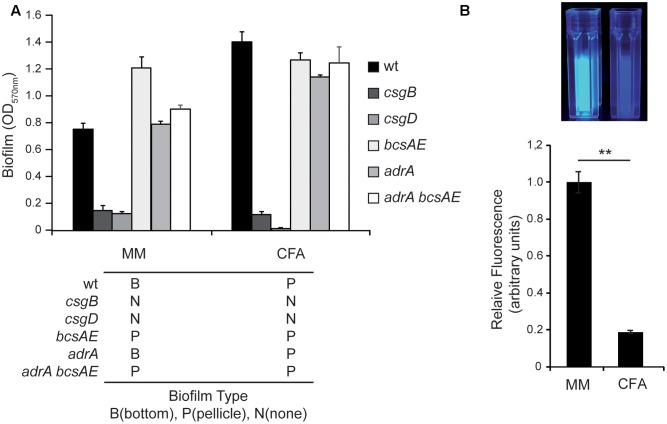
Curli but no cellulose is required for biofilm formation. **(A)** Biofilm formed by *S.* Typhimurium SV5015 (wt) and its mutant derivatives *csgB*, *csgD*, *bcsAE*, *adrA*, *adrA bcsAE* in MM and CFA. Biofilms attached to the plastic surface were stained with CV after 72 h incubation at 25°C. The type of biofilm formed by each mutant in the specified media is indicated with a B (bottom), P (pellicle), and N (no biofilm). **(B)** Production of cellulose by the wt strain in minimal media (MM) and rich media (CFA) after growing the cells for 72 h at 25°C with gentle shaking in the presence of 10 mM of CF. Fluorescence was visually observed under UV (top panel) and quantified with a fluorimeter. The results were normalized according the number of cells and expressed as arbitrary fluorescence units (bottom panel). ^∗∗^*p* < 0.005.

Having in consideration the differential expression pattern found for *csgD*, *csgB*, and *adrA* transcriptional fusions in MM and CFA, it was evaluated of whether curli and cellulose were essential promoting factors for the two types of biofilms formed in cultures grown in MM and CFA. The ability to form biofilm was studied in *csgB*, *bcsAE*, *adrA*, and *adrA bcsAE* genetic backgrounds in both media (**Figure 3**). The data from CV quantification indicate a similar pattern in cultures grown in MM and CFA. The ability to form biofilm is lost in a curli-deficient *csgB* strain independently of the culture medium used, clearly suggesting that curli is essential to form both, bottom and pellicle biofilm. Consistently, the requirement of curli to form biofilm has been previously described, being important to promote both initial cell–surface and subsequent cell–cell interactions (Steenackers et al., 2012). Interestingly, a previous report showed that although a *csgB* mutant of *Salmonella* strain ATCC 14028 exhibited a reduced ability to form biofilm, a thin ring of bacteria attached on the glass at the air–liquid interface was detected (Römling et al., 2000). In our biofilm assays no bacterial biomass attached to the plastic was detected for the *csgB* mutant strain (**Figure 3A**). It is noteworthy mentioning that strain ATCC 14028 used in the referred experiments carries a *csgD* allele that is constitutively expressed, whereas in the current report the SL1344 derivative strain used carries the native *csgD* promoter. Having in consideration that CsgD regulates biofilm promoting factors others than curli, the genetic background of the strain used may explain the described differences. In agreement with all previous statements, no biofilm was detected in a *csgD* mutant strain independently of the culture media used (**Figure 3A**).

On the other hand, a *bcsAE* mutant strain, impaired to produce cellulose, generates biofilm at similar levels than the wt strain in both, MM and CFA. These data indicate that cellulose production does not seem to be required for *Salmonella* to form biofilm under the experimental conditions tested. Two more mutant strains, *adrA* and *adrA bcsAE*, were tested and formed biofilm in MM and CFA, further corroborating that cellulose production is not essential for biofilm formation under these conditions. The spatial distribution of the biofilm generated by the different mutant strains in MM and CFA was scored. Strikingly, in MM the mutant strains *bcsAE* and *bcsAE adrA* form a pellicle biofilm instead of the bottom biofilm detected in the wt strain. These data indicate that somehow cellulose production seems to be crucial to determine the kind of biofilm that *Salmonella* produces. It may be hypothesized that cellulose production either promotes bottom biofilm formation or represses pellicle biofilm formation or both at the same time. Consequently, when cellulose production is impaired (*bcsAE* and *bcsAE adrA* mutants), a pellicle is detected in MM instead of the bottom biofilm. Moreover, from our results (**Figure 3**) it can also be inferred that cellulose is not required for the formation of a pellicle biofilm. Intriguingly, the *adrA* mutant that presumably produces very low levels of cellulose is still able to form bottom biofilm in MM.

To corroborate that there are significant differences in the amount of cellulose produced by SV5015 when growing in MM and CFA, cellulose production was monitored. Cultures were grown in the presence of calcofluor (CF), a fluorochrome that binds cellulose, and the fluorescence associated to bacterial suspensions was detected after UV exposure and quantified using a fluorimeter (**Figure 3B**). The results showed that the amount of cellulose produced in MM is much higher than in CFA (more than fivefold). The fact that an increased production of cellulose is detected in the culture conditions in which bottom biofilm is formed is in agreement with the genetic analyses data obtained indicating that the absence of the genetic determinants of cellulose causes a switch from bottom to pellicle biofilm (**Figure 3A**).

### Presence of Amino Acids Represses Cellulose Production and Promotes Pellicle Biofilm Formation

**FIGURE 4:**
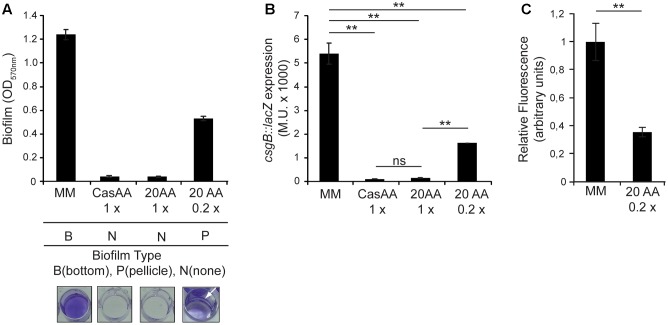
Effect of amino acids in biofilm formation. **(A)** Effect of casamino acids and a mix of 20 amino acids when added to MM on biofilm formation and **(B)** *csgB::lacZ* expression. In panel **(A)**, the type of biofilm formed in the specified media is indicated with a B (bottom), P (pellicle), and N (no biofilm). **(C)** Production of cellulose on MM and MM with a mixture of 20 amino acids (0.2×). The results were normalized according the number of cells and expressed as arbitrary fluorescence units. ^∗∗^*p* < 0.005, ns: non-significant.

So far, the data shown indicate that in CFA and MM there is a differential spatial distribution of the *Salmonella* SV5015 biofilm biomass and a differential expression pattern of the biofilm promoting factors, curli and cellulose. Which specific component of the media is responsible of the expression profile that triggers pellicle or bottom biofilm remains elusive. An evident difference in the composition of CFA and MM is the presence of abundant casamino acids in CFA, whereas no amino acids are present in MM. Several reports describe a crosstalk between amino acids metabolism and biofilm formation in *Salmonella* (Hamilton et al., 2009; Malcova et al., 2009). The influence of the presence of amino acids in biofilm formation has been explored (**Figure 4A**). Remarkably, addition of casamino acids to MM to an equivalent concentration as in CFA medium (10 g/l) causes the inhibition of the biofilm formation. Casamino acids are a complex mixture of amino acids and small peptides. Therefore, in order to determine the specific effect of pure amino acids, an equivalent concentration of a 20 amino acids-mixture was used to supplement the MM. Again, a virtually total inhibition of biofilm formation was observed. However, a dose response to the level of amino acids present was detected since addition of the amino acids-mixture at a concentration fivefold lower (0.2 x) causes a significant decrease in the biomass of the biofilm (twofold) but not full inhibition of biofilm formation. Strikingly, the type of biofilm generated changes due to the presence of amino acids (**Figure 4A**, lower panel). When amino acids were present at concentrations that allowed formation of biofilm, an obvious pellicle biofilm was detected. Since previous data suggest a correlation between the ability to generate a pellicle biofilm and low production of curli and cellulose (see *csgB* and *adrA* expression in CFA and MM, **Figures 2D,E**, **3A**), the effect of amino acids in the production of curli and cellulose was monitored. The *csgB* transcriptional expression was determined in cultures grown in the different culture media (**Figure 4B**). The expression pattern detected clearly showed that the presence of amino acids causes a downregulation of *csgB* expression. Again, a dose response to the amino acid concentration was detected. Moreover, the quantification of the cellulose production in the MM plus the 20 amino acids-mixture (0.2×) reveals a significant decrease compared to MM (**Figure 4C**). This effect is similar to the decrease observed when cells grow in CFA medium (**Figure 3B**). Altogether these results suggest that amino acids may play a crucial role in the regulation of the expression of the two biofilm promoting factors in *Salmonella*, curli and cellulose, and consequently in the spatial distribution of the generated biofilm.

### cAMP, a Physiological Sensor, Is Involved in the Spatial Distribution of the Biofilm

**FIGURE 5:**
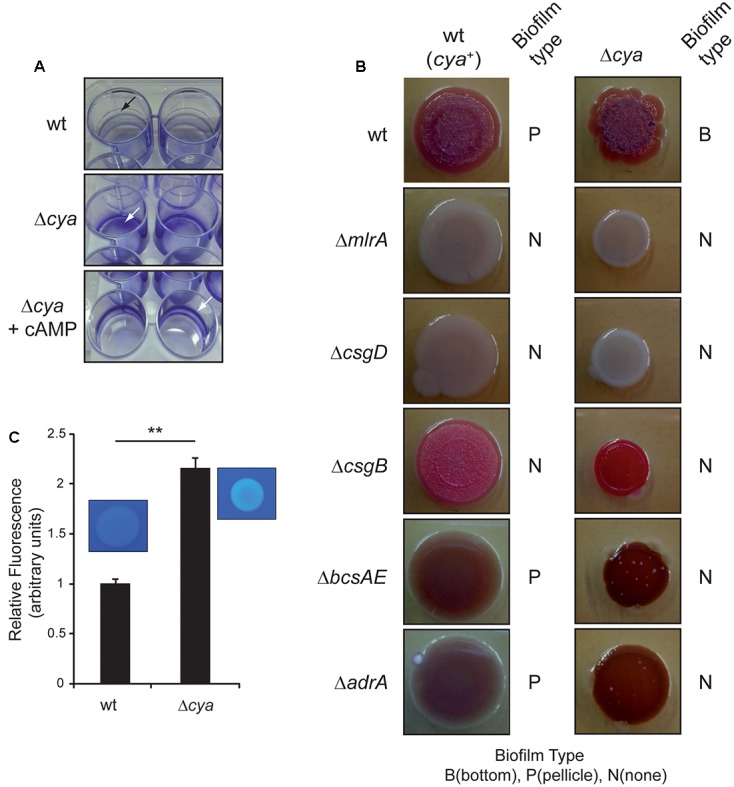
Role of cAMP in biofilm formation. **(A)** Crystal violet staining of pellicle and bottom biofilm adhered to 24-well polystyrene plate. The *cya* mutant makes a bottom biofilm in CFA at 25°C 72 h post-inoculation. The pellicle is recovered upon the addition of cAMP to the media. The arrow shows the stained biofilm. **(B)** Colony morphotype in wild type and *cya* single and double mutants on Congo red agar plates at 25°C after 96 h of incubation. The type of biofilm formed in CFA cultures is indicated with a B (bottom), P (pellicle), and N (no biofilm). **(C)** Production of cellulose by the wt and *cya* strains in CFA after growing the cells for 72 h at 25°C in the presence of 10 mM of CF. Fluorescence was visually observed under UV and quantified with a fluorimeter. The results were normalized according the number of cells and expressed as arbitrary fluorescence units. On the top panel a qualitative assessment of bacterial cellulose production determined on CF-agar plates is shown. ^∗∗^*p* < 0.005.

Genetic studies determining the effect on *Salmonella* biofilm formation of mutations in genes coding for different regulators brought an intriguing observation. A *cya* mutation causes a switch in the spatial distribution of the biofilm formed. When SV5015 strain grows in CFA, the *cya* mutation causes a shift from pellicle to bottom biofilm. Since the *cya* gene encodes the adenylate cyclase that catalyzes the synthesis of the second messenger, cAMP, our data suggest that cAMP is crucial for the formation of pellicle biofilm. This phenomenon is strictly cAMP-dependent since chemical complementation by addition of external cAMP to cultures of the *cya* strain restores the formation of the pellicle biofilm (**Figure 5A**).

To shed light on the involvement of cAMP, the multicellular morphotype of *Salmonella* SV5015 and several isogenic mutants was assessed on Congo red agar plates (**Figure 5B**). As expected, the wt strain showed a rdar (red, dry, and rough) morphotype whereas the master regulators *mlrA* and *csgD* mutants showed a saw (smooth and white) phenotype, indicating that the latests do not produce neither curli nor cellulose (Römling et al., 1998, 2000). The *csgB* mutant presented a pdar (pink, dry, and rough) phenotype as a result of production of cellulose without curli (Römling et al., 2000; Jain and Chen, 2007). Last, *bcsAE* and *adrA* mutants generate a bdar (brown, dry, and rough) morphotype characteristic of cells expressing curli but impaired in producing cellulose (Römling et al., 2000). The multicellular behavior of *cya* derivatives of all above-mentioned strains was assessed. First, the *cya* mutant showed a more pronounced rdar morphotype than the wt strain, indicating that the factors needed to display this phenotype—cellulose and curli—were being more highly expressed. The *csgB cya* mutant showed a pdar morphotype with intense pink color, suggesting higher cellulose production in the *cya* derivative. The *adrA* or *bcsAE* mutants in combination with the *cya* mutation were browner than their *cya*^+^ counterparts, indicating a higher curli production. Altogether, the results suggest that the *cya* mutation causes a general upregulation of the biofilm promoting factors, curli and cellulose. The multicellular behavior of the *cya* mutant on a *csgD* or *mlrA* background was also assessed. Interestingly, in these genetic backgrounds, the *cya* mutation did not increase the levels of curli or cellulose, clearly suggesting that the upregulation of the biofilm promoting factors in the *cya* mutant requires a functional CsgD protein. Moreover, according to previous transcriptional data, the apparently identical phenotypes shown by *mlrA* and *csgD* mutants suggest that MlrA is required for proper *csgD* expression in SV5015 strain (**Figure 2C** and Supplementary Figure S1). To corroborate the morphotype studies and confirm whether the cAMP deficiency causes an upregulation of the biofilm promoting factors, transcriptional studies were performed. The transcriptional expression of *csgD*, *csgB*, *adrA*, and *mlrA* was monitored in a *cya* mutant grown in CFA and CFA^10^ (**Figures 2C–F**). Indeed, the lack of adenylate cyclase—resulting in intracellular cAMP deficiency—produces a very drastic upregulation of *csgD* (8.3-fold) and an increase in the expression levels of *csgB* (4.9-fold). The expression of *mlrA* and *adrA* was also upregulated in a *cya* mutant, although to a much lower extent (1.9- and 1.4-fold, respectively). In high osmolarity medium, the effect of the *cya* mutation in *csgB*, *adrA*, and *mlrA* was abolished. However, *csgD* expression was almost 4.8-fold higher in the *cya* mutant than in wt after the addition of salt. Remarkably, the upregulation of *csgD* expression in the absence of cAMP is detected under osmolarity-mediated repression (high osmolarity conditions). This result suggests that osmolarity-mediated repression and the cAMP regulatory pathway are independent. No studies were performed in MM since the *cya* mutant strain is impaired to grow in this medium.

To further corroborate the effect of the *cya* mutation in the production of cellulose, both wt and *cya* strains were grown in rich media in the presence of calcofluor and the fluorescence associated to bacterial suspension was monitored (**Figure 5C**). In agreement with the morphotype analyses and the expression studies, the amount of cellulose produced by the *cya* mutant was more than twofold higher than in the wt strain.

**FIGURE 6:**
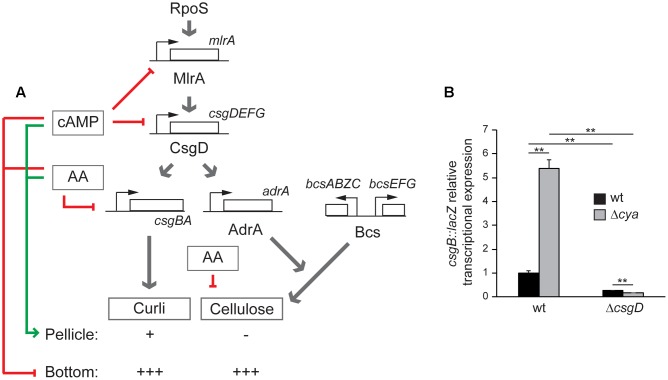
**(A)** Regulatory network controlling pellicle and bottom biofilm formation in *S. enterica* serovar Typhimurium SV5015 proposed in this work. Red arrows represent inhibitory signals and the green arrow shows positive regulation. The presence/absence and the amount of curli and cellulose is indicated with positive (from + to +++) or negative marks. **(B)** *csgB::lacZ* transcriptional expression in CFA cultures grown for 72 h at 25°C on a 24-well polystyrene plate of the indicated strains. ^∗∗^*p* < 0.005.

All the previous results suggest a model (**Figure 6**), where cAMP modulates the spatial distribution of the bacterial biofilm, promoting pellicle formation. Although a direct repressing effect of cAMP on the curli and cellulose production cannot be discarded, the fact that in the absence of *csgD* no effect by the *cya* mutation was detected (**Figure 5B**), suggests that cAMP modulates the biofilm promoting factors by repressing *csgD* expression. To further corroborate the CsgD involvement in the cAMP-mediated control of the expression of biofilm promoting factors, the *csgB* expression was monitored in presence and absence of CsgD in both wt and *cya* genetic backgrounds (**Figure 6B**). The data clearly indicate that CsgD is essential for *csgB* expression under the growth conditions assayed and that in the absence of CsgD there is not derepression of *csgB* in a *cya* mutant strain. Overall, these data further support the regulatory pathway described in which the cAMP-mediated regulation of *csgD* alters the expression of the biofilm-promoting factors such as curli. Whether cAMP-mediated repression of *csgD* is direct or indirect through *mlrA* will require further investigations. cAMP was initially described as a metabolic sensor directly involved in the carbon catabolite repression that leads to the selection of the optimal carbon source during bacterial growth (Ullmann and Monod, 1968). Studies in different bacteria demonstrated that cAMP plays a crucial role as a global regulator modulating diverse cellular processes in addition to central metabolism (Kolb et al., 1993). In this report, a relevant role of cAMP in the modulation of biofilm formation in *Salmonella* is described, suggesting that the metabolic status of the cell via cAMP can determine the multicellular behavior of *Salmonella*. The ability to form biofilm was studied in *mlrA*, *csgD*, *csgB*, *bcsAE*, and *adrA* mutant strains in a *cya* genetic background only in CFA, since no growth in MM is supported by the *cya* derivatives. The type of biofilm generated was scored (**Figure 5B** and Supplementary Figure S2). As earlier reported, the *cya* strain forms bottom biofilm in CFA. The *cya* derivatives of *mlrA*, *csgD*, and *csgB* strains are impaired to form biofilm similarly to the *cya*^+^ counterparts, indicating that MlrA, CsgD, and curli are essential for biofilm development as described above. Remarkably, the *cya bcsAE* and *cya adrA* strains, deficient in cellulose production, have lost the ability to produce bottom biofilm as compared to the *cya* mutant strain. This result suggest that cellulose production is required to form bottom biofilm. Accordingly, our model (**Figure 6A**) predict bottom biofilm formation when both curli and cellulose are produced at high levels. Data in **Figure 5B** also highlights that the *cya bcsAE* and *cya adrA* strains have lost the ability to produce pellicle biofilm as compared to the *cya*^+^ counterparts. The high levels of curli production in the *cya* strains (as indicated by the pronounced bdar phenotype) may explain the lack of pellicle biofilm in the *cya bcsAE* and *cya adrA* strains. As predicted in our model development of pellicle biofilm requires low level of curli expression.

The involvement of cAMP in biofilm formation has not been extensively studied. Although it is not valid for all the studied microorganisms, the available data suggest an overall negative role in biofilm modulation. In a former report, it was described that cAMP represses expression of type-1 fimbriae in *E. coli*, a pivotal biofilm promoting factor in this microorganism (Müller et al., 2009). Moreover, published data on *Serratia marcescens* and *Vibrio cholerae* also point to a negative role of cAMP in biofilm formation (Fong and Yildiz, 2008; Kalivoda et al., 2013). Contrarily, in a recent study performed with the uropathogenic *E. coli* UTI89, a positive role for cAMP was described (Hufnagel et al., 2016). In the present report, a negative role of cAMP in *csgD* expression and in bottom biofilm formation in *Salmonella* is described. Interestingly, cAMP has also been described as repressor of other relevant genetic elements in *Salmonella*. The *spv* virulent operon encoded by pSLT plasmid is negatively regulated by cAMP at the transcriptional level (O’Byrne and Dorman, 1994). cAMP, through its binding to CRP, was described as a transcriptional activator by binding to DNA and establishing contact with the RNA polymerase (Savery et al., 1998). Therefore, the repressing effect of cAMP is generally associated to an indirect effect. Further studies will be required to fully elucidate the molecular mechanism behind the regulatory pathway here described. In *E. coli*, the presence of PTS-transported sugar and external osmolarity have been described to affect the intracellular levels of cAMP (Saier et al., 1976; Balsalobre et al., 2006). The turnover of this second messenger depends on both synthases and phosphodiesterases (Matange, 2015). Which physiological and/or environmental signals alter the cAMP level in *Salmonella* to modulate spatial distribution of the biofilm remains to be elucidated.

## Conclusion

Biofilm formation is a complex multicellular process that involves a severe physiological cellular shift from free swimming to surface-attached bacteria. It requires important changes in the gene expression profile. Biofilm formation is triggered in response to certain environmental conditions that will activate the transition from planktonic to sessile way of life. Environmental signals that influence biofilm formation are osmolarity, pH, iron availability, oxygen tension, temperature, and nutrient composition. The response of different microorganism to the nutrient availability varies greatly. *Pseudomonas aeruginosa* form biofilm under most conditions but *E. coli* O157 only make biofilm in low-nutrient conditions and some K12 strains require amino acids to generate biofilm in minimal medium (reviewed by Davey and O’Toole, 2000). In this report, the influence of medium composition on the type of biofilm formed by *Salmonella* was studied. Under the experimental conditions assessed a shift in the spatial distribution of the biofilm was detected. Transcriptional studies indicate that the expression of *csgD*, the major regulator of the biofilm promoting factors, is affected by the growth conditions and is higher under ones promoting bottom biofilm (MM). Our data suggest a higher synthesis of curli and cellulose in MM as deduced from *csgB* and *adrA* transcriptional expression studies (**Figure 2**). Further studies will be required to fully understand the impact of the nutritional status on the regulation of biofilm formation. Our data clearly indicate the involvement of amino acids and the physiological sensor cAMP in the control of the spatial distribution of the biofilm. Although an interplay between the physiological state of the cells and biofilm formation has previously been suggested, our data indicate that this metabolic crosstalk also interferes with the spatial distribution of the generated biofilm.

## Author Contributions

SP, CM, and CB contributed in the conceptualization, formal analysis, investigation and writing. CC contributed in the investigation.

## Conflict of Interest Statement

The authors declare that the research was conducted in the absence of any commercial or financial relationships that could be construed as a potential conflict of interest.
