# Supplementary material for: Nutrient Composition Promotes Switching between Pellicle and Bottom Biofilm in Salmonella
Source: Front Microbiol. 2017 Nov 7;8:2160. doi: 10.3389/fmicb.2017.02160 (PMC5673991; doi:10.3389/fmicb.2017.02160)
Supplement: Supplementary file 1 [file Presentation_1.PDF]

**Table S1**. Strains and plasmids used in this work.

| Strain | Description | Source |
|---|---|---|
| SV5015 | *Salmonella enterica* ser. Typhimurium, SL1344 *his*$^{+}$ | 1 |
| RCT1 | SV5015 Δ*csgD* | This study |
| RCT2 | SV5015 Δ*csgB* | This study |
| RCT3 | SV5015 Δ*adrA* | This study |
| RCT4 | SV5015 Δ*bcsAE* | This study |
| RCT5 | SV5015 Δ*adrA* Δ*bcsAE* | This study |
| RCT6 | SV5015 Δ*mlrA* | This study |
| RCT7 | SV5015 *csgD::lacZ*, Km$^{R}$ | This study |
| RCT8 | SV5015 *csgB::lacZ*, Km$^{R}$ | This study |
| RCT9 | SV5015 *adrA::lacZ*, Km$^{R}$ | This study |
| RCT10 | SV5015 *mlrA::lacZ*, Km$^{R}$ | This study |
| RCT11 | SV5015 Δ*cya* | This study |
| RCT12 | SV5015 Δ*cya csgD::lacZ*, Km$^{R}$ | This study |
| RCT13 | SV5015 Δ*cya csgB::lacZ*, Km$^{R}$ | This study |
| RCT14 | SV5015 Δ*cya adrA::lacZ*, Km$^{R}$ | This study |
| RCT15 | SV5015 Δ*cya mlrA::lacZ*, Km$^{R}$ | This study |
| RCT16 | SV5015 Δ*csgD csgB::lacZ*, Km$^{R}$ Cm$^{R}$ | This study |
| RCT17 | SV5015 Δ*cya* Δ*csgD csgB::lacZ*, Km$^{R}$ Cm$^{R}$ | This study |
| | | |
| Plasmids | | |
| pKD3 | FRT Cm$^{R}$ PS1 PS2 oriR6K, Ap$^{R}$ | 2 |
| pKD4 | FRT Km$^{R}$ PS1 PS2 oriR6K, Ap$^{R}$ | 2 |
| pKD46 | $P_{BAD}$ *gam bet exo* pSC101 oriT$^{S}$, Ap$^{R}$ | 2 |
| pCP20 | *cI857* λ$P_{R}$ pSC101 oriT$^{S}$, Ap$^{R}$ Cm$^{R}$ | 2 |
| pKG136 | FRT *lacZY*+$t_{his}$ oriR6K, Km$^{R}$ | 3 |


1- Vivero, A., Baños, R. C., Mariscotti, J. F., Oliveros, J. C., Garcia-del Portillo, F., Juárez, A., et al. (2008). Modulation of horizontally acquired genes by the Hha-YdgT proteins in *Salmonella enterica* Serovar Typhimurium. J. Bacteriol. 190, 1152–1156.

2- Datsenko, K. A., and Wanner, B. L. (2000). One-step inactivation of chromosomal genes in *Escherichia coli* K-12 using PCR products. *Proc. Natl. Acad. Sci. U. S. A.* 97, 6640–6645.

3- Ellermeier, C. D., Janakiraman, A., and Slauch, J. M. (2002). Construction of targeted single copy *lac* fusions using lambda Red and FLP-mediated site-specific recombination in bacteria. *Gene* 290, 153–161.

**Table S2.** Oligonucleotides used in this work

| **Name** | **Sequence 5'-3'** | **Use** |
|---|---|---|
| CsgD_P1 | *GGGGGCAGCTGTCAGATGTGCGATTAAAAAAAGTGGAGTTTCATC*GTGTAGGCTGGAGCTGCTTC | One-step inactivation of chromosomal genes |
| CsgD_P2 | GTAACTCTGCTGCTACAATCCAGGTCAGATAGCGTTTCATGGCCCATATGAATATCCTCCTTAGT | One-step inactivation of chromosomal genes |
| CsgD_F | *GCAACATCTGTCAGTACTTC* | Genotyping |
| CsgD_R | *GCATGCAGGTTTCCGGTAGC* | Genotyping |
| mlrA_P1 | GCGTCTAAAGTTAAACCGGGACCTCGCGAGCAAGGGTGAAACGGTGTAGGCTGGAGCTGCTTC | One-step inactivation of chromosomal genes |
| mlrA_P2 | CCTTATGTTAATAAAAGGAGTATACATTAAAGCGAATTTGTTAGCTTCTGTCATATGAATATCCTCCTTAGT | One-step inactivation of chromosomal genes |
| mlrA_F | G CCGCGCCGCA ACCCGCTAC | Genotyping |
| mlrA_R | ATCACCCCGATTTTCGCTAC | Genotyping |
| CsgB_P1 | GTAATAGATAATTTTCGCTATGTACGACCAGGTCCAGGGTGACAGCGTGTAGGCTGGAGCTGCTTC | One-step inactivation of chromosomal genes |
| CsgB_P2 | CATGGTAAAACCCCCATCGGATTGATTTAAAAGTCGTAACGGTACATATGAATATCCTCCTTAGT | One-step inactivation of chromosomal genes |
| CsgB_F | CTTCATCGTAACGACGCGTT | Genotyping |
| CsgB_R | *ATTGCTGCGAATGCTGCCAC* | Genotyping |

| | | |
|---|---|---|
| adrA_P1 | TCCTCCATGCGCTCTGTTTCTATAATTTGG GAAAATTGTTTCTAAGTGTAGGCTGGAGCT GCTTC | One-step inactivation of chromosomal genes |
| adrA_P2 | GGAAAAATCAGAGGCGCTCAGTAAATCCTG AAGCCCGGCTGGACGCATATGAATATCCTC CTTAGT | One-step inactivation of chromosomal genes |
| adrA_F | CCGTAGCAAGTTTATGAGCG | Genotyping |
| adrA_R | *GAAGAACGTGGCTTCCGCGC* | Genotyping |
| bcsA_P1 | CCAGACTGACCGGGTCGTCCCAATTTAGC GTCGAGGTATAGCGCCGTGTAGGCTGGAG CTGCTTC | One-step inactivation of chromosomal genes |
| bcsE_P2 | CCGAGAGCGGCGGCGCGCCTTCAAGGAC GGCGACGCATATGAATATCCTCCTTAGT | One-step inactivation of chromosomal genes |
| bcs_F | GCACCGGTTGACGATTTAAC | Genotyping |
| bcs_R | *CCAGGGAATGATCATATTCG* | Genotyping |
| cya_P1 | TTGTACCTCTATATTGAGACTCTGAAACAG AGACTGGATGCCGTGTAGGCTGGAGCTGC TTC | One-step inactivation of chromosomal genes |
| cya_P2 | TTACGAAAAATACTGCTGCAATAGCGGCGC GTCATGATCCTGCATATGAATATCCTCCTTA GT | One-step inactivation of chromosomal genes |
| cya_F | GAGACTCTGAAACAGAGACTG | Genotyping |
| cya_R | CGGCGCGTCATGATCCTG | Genotyping |
| KT | CGGCCACAGTCGATGAATCC | Genotyping |
| LacZ_R | GATGACCTGCAAGGCGATTA | Genotyping |

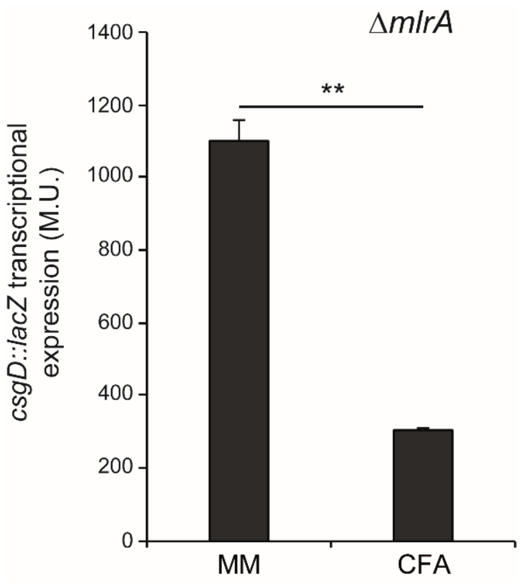


**Figure S1.** Transcriptional expression of *csgD* in a Δ*mlrA* mutant strain. Cultures were grown in the indicated media for 72 hours at 25ºC on a 24-well polystyrene plate. β-galactosidase activity was determined for three independent cultures.

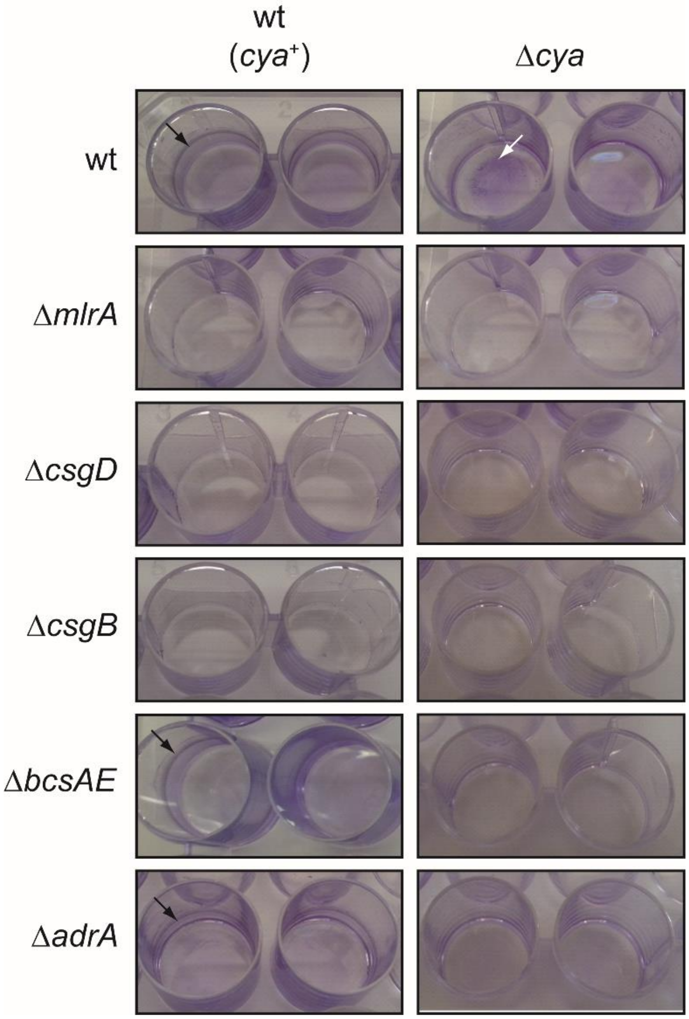


**Figure S2.** Biofilm formation of the indicated in strains grow in CFA at 25°C for 72 hours. Crystal violet staining of pellicle and bottom biofilm adhered to 24-well polystyrene plate. The arrows show the stained biofilm.
